# Editorial: Immunomodulation of MSCs in tissue repairing and regeneration

**DOI:** 10.3389/fimmu.2023.1150106

**Published:** 2023-02-13

**Authors:** Yanwen Peng, Weiqiang Li, Qunzhou Zhang

**Affiliations:** ^1^ The Biotherapy Center, the Third Affiliated Hospital, Sun Yat-sen University, Guangzhou, Guangdong, China; ^2^ Center for Stem Cell Biology and Tissue Engineering, Key Laboratory for Stem Cells and Tissue Engineering, Ministry of Education, Sun Yat-Sen University, Guangzhou, Guangdong, China; ^3^ Department of Oral and Maxillofacial Surgery & Pharmacology, School of Dental Medicine, University of Pennsylvania, Philadelphia, PA, United States

**Keywords:** MSC, immunomodulation, regenerative medicine, tissue repair, secretomes, inflammatory diseases

Due to their characteristics of easy isolation from various adult tissues, their lack of immunogenicity, and potent immunomodulatory effects on a variety of innate and adaptive immune cells, mesenchymal stem/stromal cells (MSCs) were thought to play critical roles in tissue homeostasis and tissue repair/regeneration (1). Recent studies in this field have advanced our understanding of how MSCs closely interact with their inflammatory microenvironment through their secretion of an array of bioactive factors or secretomes (1, 2), which have shed light on the elucidation of mechanisms of action of MSC-based regenerative therapy for various immune-related disorders and tissue repair (3, 4). Despite the fact that a number of preclinical studies and clinical trials support the therapeutic potentials of MSC, there are some limits that could be optimized and improved (5, 6). Our Research Topic aimed to collect articles that can provide new insights into the mechanisms involved in MSC-mediated immunomodulatory functions in the hope of optimizing their regenerative and therapeutic potentials under different disease settings. This collection includes two review articles and six research articles, which focused on the immunomodulatory properties of MSCs and MSC-based therapy for arthritis, pressure ulcer, degenerative disc disease, and noninflamed lung.

Sarsenova et al. reviewed recent advances in the approaches to enhance the immunomodulatory functions and therapeutic potentials of MSC-based therapy. Given the cross-talk between immune cells and MSCs plays an important role in MSC-based regenerative therapy, both preconditioning strategies and genetic manipulations could improve the immunomodulatory functions of MSCs. Pretreatment with pro-inflammatory cytokines, immune receptor agonists, or autophagy regulators could stimulate expression of immunomodulatory factors by MSCs. Similarly, optimization of various culture conditions for MSCs, such as changing confluency, using hypoxia or applying 3D spheroid cultures, have been reported to induce expression of genes related to immunomodulation, cell survival, proliferation, migration and homing. Genetic modification aims to overexpress or downregulate the expression of certain functional genes in MSCs so as to optimize the properties and trophic effects of MSCs in an inflammatory microenvironment, including but not limited to their proliferation, senescence, cell migration, homing, adhesion, pro-angiogenic, and survival abilities. In another review article, Che et al. provided a comprehensive review not only on the potential role of MSCs in the pathogenesis of inflammatory bowel diseases (IBDs) but also potential mechanisms of actions and current status of MSC-based regenerative therapy in the treatment of this group of chronic inflammatory disorders in the gastrointestinal (GI) tract.

Cheng et al. explored the effect of a cocktail of small molecule compounds, A-83-01, CHIR99021, and Y27632 (ACY), on the immunomodulatory functions and therapeutic efficacy of MSCs, whereby A-83–01 and Y-27632 is a specific inhibitor of TGF-β and RhoA/ROCK signaling pathway, respectively, while CHIR99021, an activator of the classic Wnt/β -catenin signaling pathway. They found that pretreatment of MSCs with ACY significantly enhanced immunosuppressive effects of MSCs on T cells and macrophages *in vitro*. Consequently, ACY-treated MSCs showed a better therapeutic potential than normal MSCs in mouse models of delayed hypersensitivity and acute peritonitis by significantly suppressing the infiltration of pro-inflammatory T cells and macrophages. Cen et al. investigated the effects of the secretomes derived from undifferentiated or osteogenically differentiated jaw periosteum-derived MSCs (JPCs) on CD14^+^ monocyte-derived dendritic cells (MoDCs). Interestingly, they found that the secretomes from osteogenically induced JPCs were able to inhibit phenotypic and functional maturation of MoDCs by destabilizing cluster formation and down-regulation of co-stimulatory surface markers. Functionally, generated MoDCs showed enhanced antigen uptake ability and suppressed CD4^+^ T cell stimulatory function and promoted CD25^+^ regulatory T cell expansion. In the research article, Katahira et al. investigated the therapeutic potential of the conditioned medium (CM) derived from the immortalized SHED (human exfoliated deciduous teeth) cell line in a cutaneous ischemia-reperfusion mouse model for pressure ulcer (PU) formation. The authors have demonstrated that CM from immortalized SHEDs exerts therapeutic effects on PU formation by promoting angiogenesis and oxidative stress resistance through vascular endothelial growth factor (VEGF) and hepatocyte growth factor (HGF). Their findings have shed light on the potential application of immortalized SHED-derived CM in the treatment of PU.

Through a conjoint analysis of weighted gene co-expression network analysis (WGCNA) and scRNA-seq, Li et al systematically analyzed the genetic and cellular components alterations of cartilage endplate (CEP) in degenerative disc disease. In this interesting article, the authors identified a group of gene signatures responsible (including BRD4, RAF1, ANGPT1, CHD7 and NOP56), highly activated immune cells (CD4^+^ T cells, NK cells, and dendritic cells), and several mesenchymal stem cells and other cellular components in degenerative CEP, thus depicted the niche atlas of different cell subpopulations, among which nucleus pulposus (NP) progenitor/mesenchymal stem cells (NPMSC) served as multipotent stem cells in CEP, exhibited regenerative and therapeutic potentials in promoting bone repair and maintaining bone homeostasis through SPP1, NRP1-related cascade reactions. Overall, this study have shown the complexity and characteristics of MSC subpopulations in CEP.

In order to better evaluate the effect of MSCs treatment of rheumatoid arthritis (RA), Lopez-Santalla et al. developed an alternative experimental model of RA by K/BxN serum transfer, which mimics many of human RA features. Their results unexpectedly showed that adipose derived MSC-based therapy couldn’t modulate the progression of K/BxN serum-transfer arthritis in mice, which was probably due to the different immune status and monocytic/macrophage balance among the different arthritic models. At last, Tynecka et al. investigated the safety of intranasal application of human adipose tissue-derived MSCs to the non-inflamed lungs. They evaluated the short-term and long-term effects of MSCs administration on lung morphology, immune responses, epithelial barrier function, and transcriptomic profiles. Their data suggested that MSCs might undergo apoptosis in the non-inflammatory microenvironment, induced low-grade inflammation in the late phases after MSC administration, subsequently leading to the re-establishment of lung tissue homeostasis

Together, these articles in this Research Topic could help us to deeply understand the targets of the MSCs-based therapy. Even though available preclinical data on the efficacy of MSCs treatment for the treatment of various inflammatory diseases are promising, the outcomes of clinical trials are unsatisfied. Under various immune-related disease conditions, MSCs’ properties and functions are plastic due to their interactions with the complicated microenvironment (1, 3). Therefore, a comprehensive understanding of the mechanisms underlying MSC-mediated immunomodulation can help us to maximize the therapeutic potential and clinical outcomes of MSC-based therapy for various immune-related/inflammatory diseases.

## Author contributions

YP, WL, and QZ contributed to conception and design of the study. YP wrote the first draft of the manuscript. All authors contributed to the article and approved the submitted version.
